# Bilateral Simultaneous Patellar Tendon Rupture in an Adolescent Athlete: A Case Report and Literature Review Highlighting the Role of Vitamin D Deficiency

**DOI:** 10.7759/cureus.109776

**Published:** 2026-05-27

**Authors:** Islam Hamarsheh, Sharo Naqar, Sarkhell Radha, Jae Rhee

**Affiliations:** 1 Trauma and Orthopaedics, Croydon University Hospital, London, GBR

**Keywords:** adolescent athlete, bilateral patellar tendon rupture, knee extensor mechanism, traumatic patellar injury, vitamin d deficiency

## Abstract

Simultaneous bilateral patellar tendon rupture is an exceptionally rare injury in the paediatric population. While bilateral patellar tendon rupture in adults is frequently associated with systemic disease or corticosteroid exposure, the few reported paediatric cases have been linked to localised tendinopathy, connective tissue disorders, or repetitive mechanical stress. This report presents a case of simultaneous bilateral patellar tendon rupture in a 15-year-old male following a rugby-related injury. Biochemical investigation revealed severe vitamin D deficiency with secondary hyperparathyroidism in the absence of systemic illness, corticosteroid use, or renal dysfunction. The patient underwent prompt bilateral surgical repair followed by structured rehabilitation and vitamin D supplementation without complications. This case represents an unusual presentation of bilateral patellar tendon rupture in an otherwise healthy adolescent and highlights the coexistence of severe vitamin D deficiency as a potentially relevant metabolic finding. Although causality cannot be inferred from a single case, recognition of potential underlying metabolic abnormalities may aid diagnostic evaluation and inform management in similar rare presentations.

## Introduction

The patellar tendon is a critical component of the knee extensor mechanism. This mechanism comprises the quadriceps muscles, which converge into a central tendon that inserts on and envelops the patella, continuing distally as the patellar tendon to act on the tibial tuberosity and facilitate knee extension [1]. Disruption of this mechanism may occur at various points along the extensor chain, most commonly involving the patella, quadriceps tendon, or patellar tendon [2]. Quadriceps tendon rupture is typically observed in individuals over the age of 40, whereas patellar tendon rupture more commonly affects men between 30 and 40 years of age [3]. Overall, across the general population, patellar tendon rupture occurs less frequently than quadriceps tendon rupture or patellar fracture [4].

Patellar tendon rupture is a rare injury in children, with limited data available regarding its frequency and outcomes. In a case series of 71 paediatric patients presenting with injuries to the knee extensor mechanism, patellar tendon rupture accounted for approximately seven percent of cases [5]. In paediatric individuals, bone avulsion injuries, such as patellar sleeve fractures, are more commonly encountered [6]. In contrast, isolated tendon rupture without associated avulsion is exceedingly uncommon, with only a few cases reported in the literature.

Although trauma represents the most frequent cause of patellar tendon rupture, spontaneous ruptures have been associated with underlying metabolic or rheumatologic disorders [7,8]. Additional risk factors include exposure to quinolone antibiotics and corticosteroid therapy [9,10].

Bilateral patellar tendon rupture is most frequently linked to corticosteroid use in adults and has also been reported in association with systemic lupus erythematosus, chronic renal failure, rheumatoid arthritis, and Osgood-Schlatter disease [11]. To the authors’ knowledge, only four cases of bilateral patellar tendon rupture have been reported in paediatric patients to date, and coexistence with severe vitamin D deficiency has not been previously described [12-15]. Documentation of this case expands the limited paediatric literature on bilateral patellar tendon rupture, emphasises the importance of considering metabolic bone abnormalities in unusual extensor mechanism injuries, and may help inform diagnostic evaluation and multidisciplinary management in similar rare presentations.

## Case presentation

In October 2025, a 15-year-old boy presented to the Accident and Emergency Department at Croydon University Hospital, London, United Kingdom, with acute bilateral knee pain and swelling following a rugby-related injury sustained on the same day. He reported a direct lateral impact during a rugby tackle, followed by a fall onto both knees. Immediately after the injury, he developed bilateral anterior knee pain, swelling, and difficulty mobilising. He was able to partially bear weight but reported significant pain with knee movement. His medical history included seasonal asthma managed with salbutamol inhalers and a previous anterior inferior iliac spine avulsion fracture sustained while running one year earlier. There was no history of chronic systemic illness, renal disease, inflammatory arthritis, corticosteroid exposure, fluoroquinolone use, or previous knee surgery.

On examination, both knees were swollen with palpable effusions. The patient was unable to actively extend either knee, and a palpable gap was identified in both patellar tendons. Based on the mechanism of injury and clinical findings, the initial differential diagnoses included bilateral patellar tendon rupture, patellar sleeve fracture, tibial tubercle avulsion injury, patellar fracture, quadriceps tendon injury, and associated intra-articular knee injury, including ligamentous injury. Plain radiographs demonstrated bilateral patella alta with soft-tissue swelling and disruption of the anterior soft-tissue planes (Figure 1).

**Figure 1:**
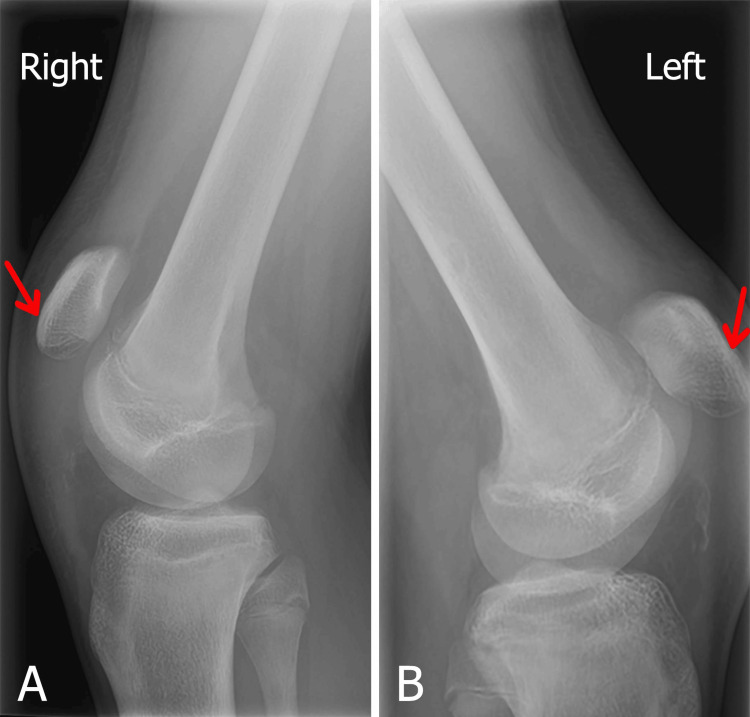
Bilateral lateral knee radiographs at presentation. (A) Right knee demonstrating patella alta (red arrow) with anterior infrapatellar soft-tissue swelling. (B) Left knee showing similar patella alta (red arrow) with disruption of the anterior infrapatellar soft-tissue planes. All radiographic images are original and were obtained by the authors for this case.

Magnetic resonance imaging (MRI) confirmed bilateral proximal patellar tendon ruptures. Sagittal proton-density fat-suppressed sequences demonstrated complete disruption of the proximal patellar tendons at their insertion on the inferior poles of the patellae, with proximal tendon retraction and surrounding soft-tissue oedema of high signal intensity at the rupture sites. On the right, the tendon was curled proximally with a 15 mm gap. On the left, the gap measured approximately 16 mm. Distal tendon lengths measured approximately 4.7 cm bilaterally. In addition, diffuse, symmetrical metaphyseal bone marrow oedema of high signal intensity was observed in the distal femora, proximal tibiae, and fibulae (Figures 2, 3). A pelvic radiograph obtained in January 2024 showed widened pelvic physes, raising suspicion for metabolic bone disease such as vitamin D deficiency.

**Figure 2:**
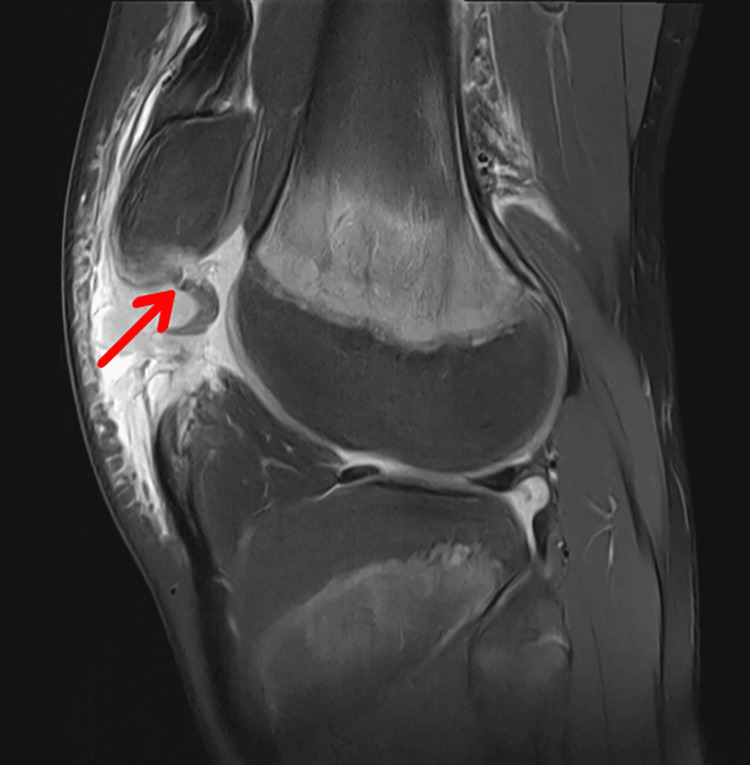
Sagittal proton-density fat-suppressed MRI of the right knee. Red arrow indicates proximal patellar tendon rupture at the inferior pole of the patella, with tendon discontinuity, proximal tendon retraction, and surrounding soft-tissue oedema of high signal intensity. All radiographic images are original and were obtained by the authors for this case.

**Figure 3:**
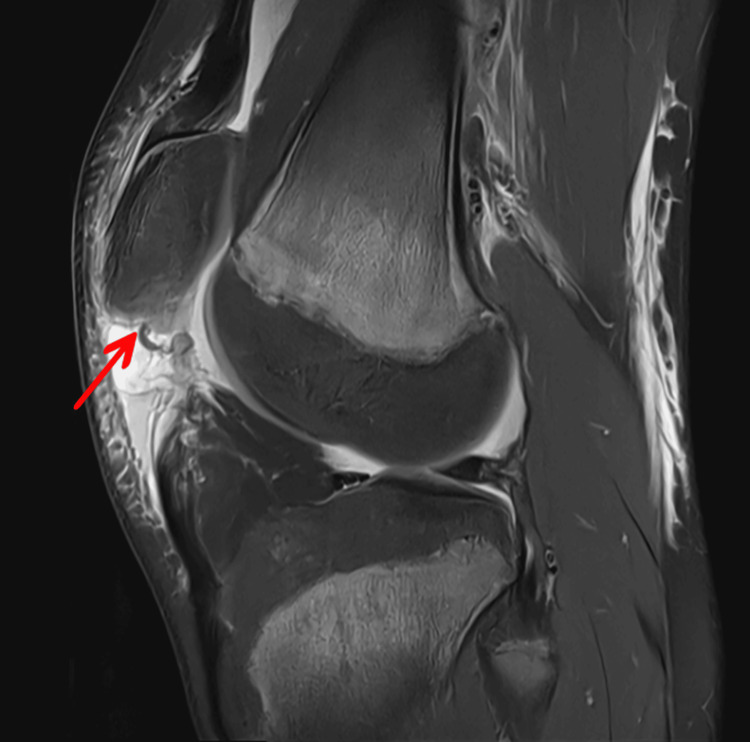
Sagittal proton-density fat-suppressed MRI of the left knee. Red arrow indicates proximal patellar tendon rupture at its insertion on the inferior pole of the patella, with tendon discontinuity, proximal tendon retraction, and surrounding soft-tissue oedema of high signal intensity. All radiographic images are original and were obtained by the authors for this case.

Haematologic and bone metabolism studies revealed severe vitamin D deficiency (<11 nmol/L) and markedly elevated parathyroid hormone (PTH) (89.3 pmol/L), with normal adjusted calcium, phosphate, and renal function (Table 1). Alkaline phosphatase was elevated at 591 U/L. This was considered consistent with increased bone turnover in the context of severe vitamin D deficiency and secondary hyperparathyroidism, although alkaline phosphatase is a non-specific marker and was interpreted alongside the broader bone and liver profile.

**Table 1:** Haematologic and bone metabolism laboratory results showing severe vitamin D deficiency and secondary hyperparathyroidism.

Parameter	Result	Reference range
Haemoglobin	131 g/L	120-160
White cell count	5.7 x 10^9/L	4-11
Calcium	2.18 mmol/L	2.2-2.7
Adjusted calcium	2.24 mmol/L	2.2-2.7
Phosphate	1.30 mmol/L	0.9-1.8
Alkaline phosphatase	591 U/L	60-425
Alanine aminotransferase	6 U/L	0-52
Albumin	37 g/L	35-50
C-reactive protein	13 mg/L	0-5
Vitamin C	26.9 µmol/L	26.1-84.6
Thyroid stimulating hormone (TSH)	1.47 mlU/L	0.51-4.3
Free thyroxine (fT4)	9.7 pmol/L	7.4-15.6
Parathyroid hormone (PTH)	89.3 pmol/L	1.1-6.9
Total 25OH vitamin D	<11 nmol/L	50-174

Dietetic review identified a body mass index (BMI) of 17.0 kg/m² and suboptimal nutritional intake, with an estimated oral intake of approximately 1200 kcal/day and 30 g protein/day, below estimated requirements for age and weight. Dietary history was notable for frequent omission of breakfast, variable school meals, and limited dairy intake.

Surgical repair was performed under general anaesthesia. Fixation was achieved using two longitudinal transosseous tunnels, each measuring 2.0 mm, drilled in each patella. Two size five Ethibond whipstitch sutures were placed in each distal tendon, passed through the tunnels, and tied proximally. The repair was reinforced circumferentially around the patella in a cerclage fashion for added stability. Intraoperative testing through 90° of knee flexion confirmed satisfactory tension and secure tendon apposition bilaterally.

Postoperatively, both limbs were immobilised in full extension with cricket-pad splints. Physiotherapy began on postoperative day one, progressing from assisted to independent ambulation by day seven, with good quadriceps activation and minimal discomfort. Vitamin D supplementation was initiated under paediatric supervision. Two weeks later, the splints were replaced with hinged knee braces locked at 0-30° of flexion, with flexion gradually increased every two weeks. By the third postoperative week, the wounds had healed, and the patient was fully weight-bearing with a frame walker.

Follow-up demonstrated gradual functional improvement under combined orthopaedic, physiotherapy, paediatric, and dietetic care. By late December 2025, the patient reported no pain or discomfort, was able to manage stairs, walked with a smoother gait pattern, sat with both knees flexed to 90°, and achieved active knee flexion to approximately 100°. The braces had progressed to allow 0-120° of flexion. At orthopaedic follow-up in January 2026, the braces were discontinued. The patient was walking without braces at home and had good bilateral knee extension without pain. Paediatric and dietetic follow-up continued for micronutrient deficiency with ongoing cholecalciferol and multivitamin supplementation, dietary advice to increase calcium intake, and planned biochemical monitoring. At the fracture clinic review in April 2026, he remained engaged in physiotherapy rehabilitation and was advised to continue physiotherapy rehabilitation for muscle strengthening pending further review by the operating team. No wound complications, infection, extensor mechanism failure, or re-rupture were reported during the documented follow-up period.

## Discussion

Epidemiology and pathophysiology of bilateral patellar tendon rupture in the paediatric population

Bilateral rupture of the patellar tendon in the paediatric population is exceptionally uncommon. In contrast to adults, in whom systemic conditions are well-established predisposing factors [10], true bilateral tendon rupture in otherwise healthy children or adolescents remains exceedingly rare, typically results from degenerative tendinopathy secondary to repetitive trauma [16].

The rarity of this injury in skeletally immature individuals is largely attributed to the unique biomechanical characteristics of the paediatric knee. In children, the cartilaginous patella and open physes render the osteochondral structures relatively weaker than the surrounding tendons. Consequently, most extensor mechanism injuries in this age group manifest as tibial tubercle avulsion fractures rather than intratendinous ruptures [17]. Similarly, patellar sleeve fractures, which represent 57-72% of paediatric patellar fractures, are more common than tendon substance rupture [6].

Experimental studies have shown that forces of up to 17.5 times body weight are required to rupture a normal human patellar tendon [18], making spontaneous rupture uncommon in the absence of underlying pathology.

Histopathological studies of ruptured tendons consistently reveal degenerative alterations, including collagen disorganisation, tenocyte necrosis, and vascular proliferation, indicating that rupture may occur when cumulative microtrauma or systemic factors contribute to critical structural compromise [19]. These degenerative processes may be exacerbated by inflammatory or metabolic disorders or by corticosteroid therapy, which reduces collagen synthesis and tensile strength [20]. In adolescents, repetitive microstress from sports-related activities may further predispose to tendinous weakening [21].

Comparison with previously reported paediatric cases

Muratli et al. described a nine-year-old boy with sequential bilateral mid-substance patellar tendon ruptures, the first case of its kind reported in the literature [12]. Histological analysis revealed structurally normal tendon tissue, suggesting that mechanical overloading of immature collagen was the primary mechanism of injury. Moretti et al. subsequently reported bilateral proximal avulsion in a 13-year-old patient with Ehlers-Danlos syndrome, highlighting how collagen synthesis disorders can markedly compromise tendon integrity [13]. Kim et al. later described a healthy 12-year-old athlete who sustained concurrent bilateral ruptures at the inferior patellar poles, attributing the injury to repetitive microtrauma and insertional stress [14]. More recently, Yonga et al. reported bilateral rupture in a 14-year-old basketball player with Osgood-Schlatter disease, in which persistent traction apophysitis and localised microinflammation may lead to progressive degeneration at the tibial tubercle attachment [15,22]. 

Considering these mechanisms, the bilateral tendon rupture observed in the present case may reflect a multifactorial process in which repetitive sports-related mechanical loading and severe vitamin D deficiency coexisted as potentially relevant factors. The presence of metaphyseal bone marrow oedema and physeal widening on MRI may be consistent with disturbed mineralisation in the setting of severe vitamin D deficiency. However, these findings should be interpreted cautiously and cannot establish a direct causal pathway to tendon rupture [23].

Other potential contributors should also be considered in this case. Connective tissue disorders, including Ehlers-Danlos syndrome, may compromise collagen integrity and have been reported in association with bilateral patellar tendon rupture in paediatric patients [13]. In addition, tendinopathy may develop along a pathological continuum in which repetitive mechanical loading leads to cumulative microtrauma, failed tendon adaptation, and progressive changes in collagen organisation and tendon structure. When recovery is inadequate, degenerative matrix changes, neovascularisation, and neural ingrowth may occur, contributing to pain and impaired tendon function [24]. In the present case, there was no history of systemic inflammatory disease, renal dysfunction, corticosteroid exposure, constitutional symptoms, lymphadenopathy, hepatosplenomegaly, or recurrent tendon injury. Paediatric and metabolic bone assessment identified severe vitamin D deficiency with secondary hyperparathyroidism and clinical features consistent with rickets, while malabsorption and nutritional insufficiency were also considered as contributing factors. However, a formal rheumatological or genetic assessment was not performed. Therefore, subclinical connective tissue disease or genetic predisposition cannot be definitively excluded.

Role of vitamin D deficiency in tendon health and integrity

Vitamin D plays an important role not only in bone metabolism but also in maintaining the structural and metabolic integrity of skeletal muscles, ligaments, and tendons. Its biological effects are mediated through the vitamin D receptor (VDR), which may influence collagen synthesis, extracellular matrix turnover, and inflammatory activity within musculoskeletal tissues [25-28]. It has been demonstrated that 1-α-hydroxylase and the VDR are expressed in human tenocytes, indicating that these cells have the ability to utilise inactive forms of vitamin D and interact with it directly. When tenocytes were treated with dexamethasone to induce cell injury, vitamin D co-treatment restored cell proliferation in a dose-dependent manner, improved the expression of tenocyte-related markers such as collagen type I, and reduced reactive oxygen species production [29]. Although these findings are mechanistically compelling, in vitro models do not fully replicate the complex biomechanical and systemic environment in which tendon pathology develops in vivo; therefore, caution is warranted when extrapolating these findings to clinical practice.

Experimental and histopathological studies suggest that vitamin D deficiency may alter collagen organisation and proteoglycan composition within tendons and periarticular structures. Dean et al. reported that vitamin D modulates matrix metalloproteinase activity in matrix vesicles and that deficiency may promote catabolic enzyme expression, predisposing to extracellular matrix degradation [25]. In a porcine model of hyperlipidaemic microswine, vitamin D deficiency was associated with increased inflammation, fatty infiltration, and architectural disorganisation in the quadriceps muscle, patellar tendon, and collateral ligaments. These findings suggest that vitamin D status may influence the structural and metabolic homeostasis of tendons and ligaments. The same study also reported differences in triggering receptor expressed on myeloid cells-2 (TREM-2) activity and macrophage localisation across knee joint tissues, indicating that vitamin D may have a role in regulating local inflammatory responses and supporting tissue protection [30].

Vitamin D supplementation for periods exceeding one week, at doses of at least 2000 IU/day, has been shown to attenuate exercise-induced muscle damage and inflammation. Adequate vitamin D status also supports muscular function during exercise and may accelerate recovery through reductions in pro-inflammatory cytokines [31]. Recent research has further highlighted a potential association between hypovitaminosis D and soft-tissue injuries. Frank et al. reported that 47.2% of collegiate athletes were vitamin D insufficient and 17.6% were deficient; moreover, each 5 ng/mL (≈12.5 nmol/L) decrease in serum vitamin D level was associated with a 13% increase in the odds of musculoskeletal injury [32]. Similarly, Ammerman et al. found that among young female patients presenting with lower-extremity musculoskeletal complaints, 54.6% of those with tendon or muscle injuries had low vitamin D levels, a proportion significantly higher than that observed in other injury types [33]. Collectively, these findings suggest a possible association between suboptimal vitamin D status and musculoskeletal injuries involving both bone and soft tissues. Consistent with this, a significant association between vitamin D deficiency and lateral epicondylitis in young adults (mean age approximately 22 years) has been reported [34]. However, although the study excluded participants with rheumatologic, endocrine, or autoimmune disorders, other potential confounding risk factors for lateral epicondylitis were not assessed, thereby limiting causal inference. Furthermore, these studies are observational in design and therefore cannot establish causality; residual confounding from factors such as physical activity level, dietary intake, sun exposure, and baseline tendon integrity cannot be excluded.

The patient’s history of asthma provides an additional clinical context in which to consider the broader relevance of vitamin D deficiency. Observational studies have reported that children with asthma have lower serum 25-hydroxyvitamin D levels than non-asthmatic children [35]. However, associations between vitamin D concentration and asthma incidence, asthma control, and lung function remain inconsistent. Therefore, although the coexistence of asthma and vitamin D deficiency in the present case is noteworthy, no case-specific inference can be made regarding the relevance of vitamin D status to the patient’s asthma control or respiratory function, as these parameters were not assessed.

Surgical and rehabilitation considerations

Timely surgical repair remains the cornerstone of management for bilateral patellar tendon rupture. A recent systematic review of 45 bilateral cases involving 90 tendons reported that primary repair using end-to-end suturing, transosseous tunnels, or suture anchors was the preferred approach in most instances [3]. Augmentation techniques, such as cerclage wiring or autograft reinforcement, were frequently employed when tendon quality was poor, while reconstruction was reserved for chronic or severely degenerated cases. Although these findings are derived predominantly from adult populations, they underscore the importance of early anatomical repair with strong fixation, a principle equally applicable to adolescents when the native tendon tissue remains viable.

Among the four previously reported paediatric cases of bilateral patellar tendon rupture, all were managed surgically with excellent long-term outcomes. Muratli et al. performed primary end-to-end repair reinforced with cerclage wiring and Achilles tendon allograft augmentation, achieving full range of motion (ROM) and strength at one year [12]. Moretti et al. utilised suture-anchor repair with cerclage protection, resulting in complete recovery and return to activity within ten weeks [13]. Kim et al. employed non-absorbable sutures with titanium anchors followed by six weeks of immobilisation and structured rehabilitation, restoring a full range of motion (ROM) (0-130°) by four months and returning to sport at one year [14]. Yonga et al. used suture-anchor fixation reinforced with U-staples and hinged-brace rehabilitation, achieving near-normal patellar height and a Tegner-Lysholm score of 96 at five-year follow-up [15]. Table 2 provides a summary comparison of the four reported cases, including patient age, rupture site, surgical technique, and reported outcome. 

**Table 2:** Summary of the reported paediatric cases of bilateral patellar tendon rupture. Data adapted from Muratli et al. [12], Moretti et al. [13], Kim et al. [14], and Yonga et al. [15]. ROM: range of motion.

Author (year)	Age	Site	Surgical technique	Rehabilitation	Reported outcome
Muratli et al. [12]	9	Mid-substance	Primary end-to-end repair + cerclage + Achilles-tendon allograft	Cast six weeks. Gradual ROM to 120-130°	Full ROM and strength at one year; no complications
Moretti et al. [13]	13	Proximal insertion	Suture-anchor repair + temporary cerclage	Brace locked 0° for one week. ROM 0-90° by three weeks	Full recovery by 10 weeks; resumed activity, no recurrence
Kim et al. [14]	12	Inferior pole	Non-absorbable sutures + titanium anchors	Extension cast for six weeks. Progressive rehabilitation	ROM 0-130° by four months; return to sport at one year
Yonga et al. [15]	14	Distal enthesis	Suture-anchor + U-staple reinforcement	Hinged brace. Staged flexion to 120° by six weeks	Full ROM; no complications or recurrence

Limitations

This report is limited by its single-case design, which precludes causal inference between vitamin D deficiency and patellar tendon rupture, and by the incomplete assessment of contextual factors that may influence vitamin D status. Although the dietetic review identified suboptimal nutritional intake and limited dietary calcium intake, detailed information regarding sunlight exposure and socioeconomic context was not systematically recorded. In addition, histopathological analysis of the ruptured tendons was not performed; therefore, the proposed mechanism of subclinical tendon degeneration remains speculative and cannot be directly confirmed at the tissue level. Nevertheless, the rarity of simultaneous bilateral patellar tendon rupture in adolescents highlights the clinical value of reporting this case and supports further investigation into potential metabolic contributors to tendon vulnerability.

## Conclusions

Bilateral patellar tendon rupture in adolescents without underlying systemic disease is exceptionally rare. This case illustrates the coexistence of repetitive mechanical loading and severe vitamin D deficiency as potentially contributory factors in an adolescent with bilateral patellar tendon rupture. Although causality cannot be established from a single case, this presentation highlights the need for further investigation into the potential interaction between vitamin D deficiency and mechanical stress in paediatric tendon pathology.
